# Persistent organic pollutants in Antarctic notothenioid fish and invertebrates associated with trophic levels

**DOI:** 10.1371/journal.pone.0194147

**Published:** 2018-04-11

**Authors:** Fung-Chi Ko, Wei-Ling Pan, Jing-O Cheng, Te-Hao Chen, Fu-Wen Kuo, Shu-Ji Kao, Chih-Wei Chang, Hsuan-Ching Ho, Wei-Hsien Wang, Li-Sing Fang

**Affiliations:** 1 National Museum of Marine Biology and Aquarium, Pingtung, Taiwan; 2 Institute of Marine Biology, National Dong Hwa University, Pingtung, Taiwan; 3 State Key Laboratory of Marine Environmental Science, Xiamen University, China; 4 Department of Marine Biotechnology and Resources, National Sun Yat-sen University, Kaohsiung, Taiwan; 5 Departmentof Leisure and Sport Management, Cheng Shiu University, Kaohsiung, Taiwan; National Taiwan Ocean University, TAIWAN

## Abstract

Notothenioid fish and invertebrate samples from Antarctica were collected in the austral summer of 2009, and analyzed for persistent organic pollutants (POPs), including polycyclic aromatic hydrocarbons (PAHs), organochlorine pesticides (OCPs), and polybrominated diphenylethers (PBDEs), as well as δ^13^C and δ^15^N stable isotopes for trophic level determination. In this study, the POP levels in the Antarctic biota samples were found to be ranked in the following order: OCPs > PAHs >> PBDEs. The POP levels in notothenioid fish and krill correlate to trophic levels; however, the POP concentrations in intertidal benthic invertebrates are higher than in notothenioid fish implying that specific biogeochemical factors may affect bioaccumulation in the Antarctica ecosystem. Biomagnification of POPs may have a smaller role than bioconcentration in Antarctica environment. In addition to the source, transport, exposure, and absorption for each group of POPs in the short food chain in Antarctica, the biological variation among species, interaction habitats, diet and metabolism are also factors for future studies on contaminant bioaccumulation.

## Introduction

Persistent organic pollutants (POPs) are ubiquitous anthropogenic chemicals that can transfer over long distances and bioaccumulate through food webs, causing health risks to wildlife and humans. Although many POPs such as organochlorine pesticides (OCPs), and polybrominated diphenyl ethers (PBDEs) have been banned, they still continue to be reported at toxic concentrations in organisms, even in the Polar Regions [1, 2, 3]. Scientists have found the presence of POPs in samples from Antarctica since the 1960s [4, 5, 6, 7]. The extremely low temperatures in Antarctica enhance the low volatility and low degradation rate of POPs [8], allowing the polar environment to serve as a global sink for persistent pollutants [9, 10, 11]. Cold-adapted organisms in Antarctic have slower metabolism, resulting in low rates of growth and reproduction [12]. Most polar organisms depend lipids for energy storage, and thus may accumulate more POPs during a lifespan [13], leading to the efficient transfer and accumulation of POPs in the food chain [14, 15]. The Antarctic food chain is fairly simple and short [8], so fishes and invertebrates are key sources of POP bioaccumulation in the Antarctic marine ecosystem. Notothenioid fish (suborder Notothenioidei), an endemic coastal demersal group, includes six dominant families in terms of diversity and biomass [16, 17]. Notothenioid fish constitute an important link in the Antarctic marine food chain, since they prey on a variety of pelagic, benthic and epibenthic organisms, and they are preyed upon by squids, higher trophic level fishes, penguins, sea birds, and marine mammals such as seals and whales [18]. Therefore, studying POP bioaccumulation in notothenioid fish is important to understand the transfer and distribution of POPs in the Antarctica food web.

When entering an organism, different POPs present different degradation rates and bioaccumulation patterns among tissues, depending on their physiochemical properties and the metabolic system involved [19,20]. However, contaminant distribution patterns in tissues are not only affected by the POP chemical structure and its major metabolites, but also by the biology and ecology of organism in question [21, 22].

Due to the sampling limitations, the sample sizes in this study were low. However, our results can be taken as indicative of the POP baseline of Antarctic fishes and invertebrates. The objectives of this study were to determine and compare the occurrence, distribution, and profiles of target POPs (including PBDEs, PAHs, and OCPs) in muscle, liver, egg, and stomach of Antarctic fishes and the invertebrates. Furthermore, δ^13^C and δ^15^N stable isotopes measurements were used to investigate the correlation between POP levels and trophic levels and to model bioaccumulation and biomagnification through the Antarctic food chain.

## Materials and methods

### Collection of samples

**Fig 1:**
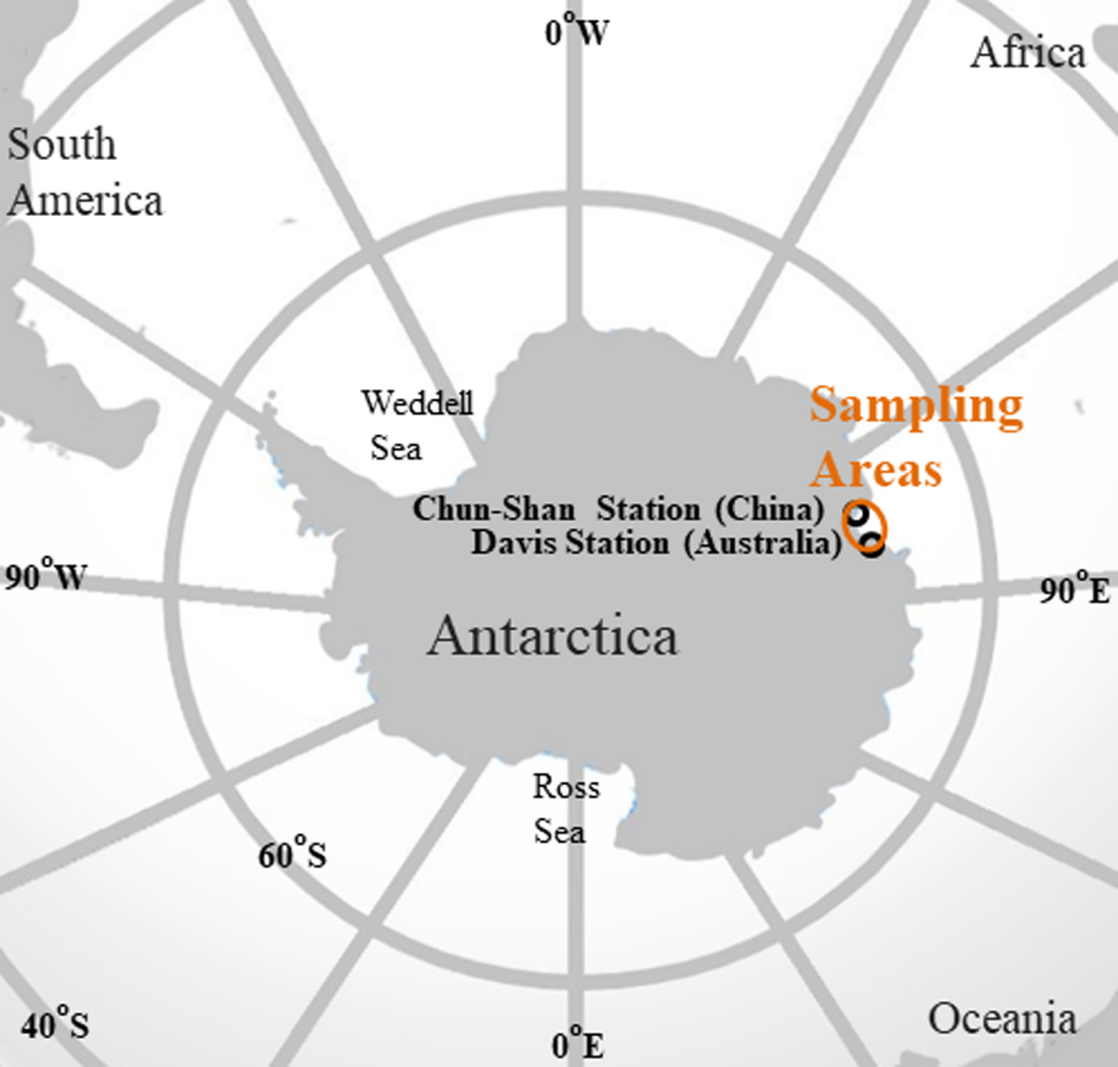
Fish and invertebrate samples were collected from the coastal areas between Chun-Shan station and Davis station adjacent waters in eastern Antarctica.

Fishes and invertebrates were collected from the coastal areas between Chun-Shan Station (-69.371483°, 76.385906°) and Davis Station (-68.583043°, 77.921333°) adjacent waters in eastern Antarctica (Fig 1) in the austral summer of December 2009 –January 2010. The collected samples include six species fish: *Chaenocephalus aceratus*, *Chionodraco rastrospinosus*, *Gobionotothen gibberifrons*, *Gymnodraco acuticeps*, *Pagothenia borchgrevinki*, *Pseudotrematomus bernacchii*, and nine species of invertebrates: Gammaridae gn. sp., *Euphausia superba*, *Nacella concinna*, *Amauropsis* sp., *Yoldia* sp., *Ophionotus victoriae*, *Sterechinus neumayeri*, *Sterechinus* sp., and Salpidae gn. sp. (S1 Table). The studied Antarctic species are demersal or bathypelagic. All tissue samples were stored immediately at -20°C until analysis.

### Chemical analysis

The analytical methodology used is described in Ko et al., 2014 [23, 24]. Briefly, dried tissue aliquots were homogenized with sodium sulfate. The homogenate was extracted using accelerated solvent extraction (ASE-300, Dionex, USA). A fraction of the resulting extract was used for the determination of lipid content by gravimetry. The remaining extract was further purified by fractionation on a multilayer silica gel column for instrumental analysis.

Detection and quantification of analytes was carried out using a Varian CP-3800 gas chromatography coupled with a Model 320 triple-quadrupole mass spectrometer (Varian, USA) equipped with electron ionization (EI) source for PAH and OCP analysis; negative chemical ionization (NCI) source for PBDE analysis. Both sources were in the selected ion monitoring (SIM) mode. A 30m x 0.25mm x 0.25μm, DB-5 capillary column (J&W Scientific, USA) was used for the determination of PAHs and OCPs. A VF-5MS (10m x 0.53mm x 0.25um film thickness) rapid capillary column was used for the determination of PBDE congeners. The investigated compounds (Table 1) include 24 PAHs, 11 OCPs, and 17 PBDEs.

**Table 1:** The investigated POPs (24 PAHs, 11 OCPs, and 17 congeners of PBDEs) and the method detection limit (MDL) of each compound.

PAHs	MDL (ng)	OCPs	MDL (ng)	PBDE congeners	MDL (ng)
**Acenapthylene,**	0.51	α-HCH	0.19	BDE15	0.07
**Acenaphthene**	1.70	β-HCH	0.64	BDE17	0.03
**Fluorene**	2.19	γ-HCH	0.82	BDE28	0.06
**Dibenzothiophene**	1.02	δ-HCH	0.30	BDE71	0.06
**Phenanthrene**	3.03	HCB	0.09	BDE47	0.02
**Anthracene**	0.65	*o*,*p'*-DDE	0.04	BDE66	0.55
**4,6dimethyldibenzothiop**	0.39	*p*,*p'*-DDE	0.08	BDE100	0.02
**Fluoranthene**	1.20	*o*,*p'*-DDD	0.07	BDE99	0.03
**Pyrene**	0.92	*p*,*p'*-DDD	0.08	BDE85	0.01
**Retene**	0.21	*o*,*p'*-DDT	0.07	BDE154	0.02
**Benzo[*a*]fluorine**	0.08	*p*,*p'*-DDT	0.18	BDE153	0.02
**Benzo[*b*]fluorine**	0.13			BDE138	0.02
**Benz[*a*]anthracene**	0.50			BDE183	0.03
Chrysene/Triphenylene*	0.87			BDE190	0.03
**Benzo[*b*]fluoranthene**	0.82			BDE203	0.09
**Benzo[*k*]fluoranthene**	0.57			BDE205	0.05
**Benzo[*e*]pyrene**	0.85			BDE206	0.40
**Benzo[*a*]pyrene**	1.15				
**Perylene**	0.75				
**Indeno[*1*,*2*,*3-c*,*d*]pyrene**	0.36				
**Dibenz[*a*,*h*]anthracene**	0.52				
**Benzo[*g*,*h*,*i*]perylene**	0.77				
**Coronene**	0.28				

* Chrysene and triphenylene were not chromatographically resolved, thus the MDL was pooled and the sum of these compounds was used in the data analysis and discussion.

Laboratory protocol and quality assurance/quality control (QA/QC) followed guidelines described by Ko et al., 2014 [23, 24]. Method validation was performed using the NIST intercomparison exercise for the determination of persistent organic compounds in whale blubber samples (NIST SRM 1945). Surrogate recovery ranged from 88% to 101%. Method detection limits (MDLs) were set as the average plus three times the standard deviation of ten blank technical replicates (Table 1). Spiked matrices were recovered within the acceptable ranges (78–110% of the spiked analytes). After freeze-drying, subsamples of the muscle were measured for stable isotopes (δ^13^C and δ^15^N). Clean up was performed in a test tube containing 100 mg of sample and 4 mL of cyclohexane. The mixture was shaken for an hour, then centrifuged for separation and dried at 50°C for 48 hr. Purified samples were analyzed using a Thermo Finnigan Delta plus Advantage isotope ratio mass spectrometer. Pee Dee Belemnite and atmospheric nitrogen (IAEA-TECDOC-825, 1995) were used as standards for quantification of δ^13^C and δ^15^N, respectively. Isotopic values were expressed in parts per thousand relative to standards: δ^13^C or δ^15^N = [(R_sample_/R_standard_) -1] x 10^3^, where R = δ^13^C or δ^15^N. Based on replicate measurements of internal laboratory standards, experimental precision is ±0.15‰ and ±0.20‰ for δ^13^C and δ^15^N, respectively.

### Data analysis

The trophic level (TL) of a species is a function of the ^15^N content in their tissue (Post et al., 2000). The reference first trophic level may vary due to factors such as sampling site and trophic structure. In this study, the TLs were estimated by the classic formula reported in Post et al., (2000): TL_species_ = [(δ^15^N_secondary consumer_ - δ^15^N_primary consumer_)/3.4] + 2, where δ^15^N_primary consumer_ was shrimp in this study and 3.4‰ was a constant per-trophic-level fractionation [25]. Regression analysis was used to estimate the best fit [26] between POP concentration (log transformed) and trophic levels in each species according to the following simple linear regression: log [POP] = a + (b x TL).

## Results and discussions

**Fig 2:**
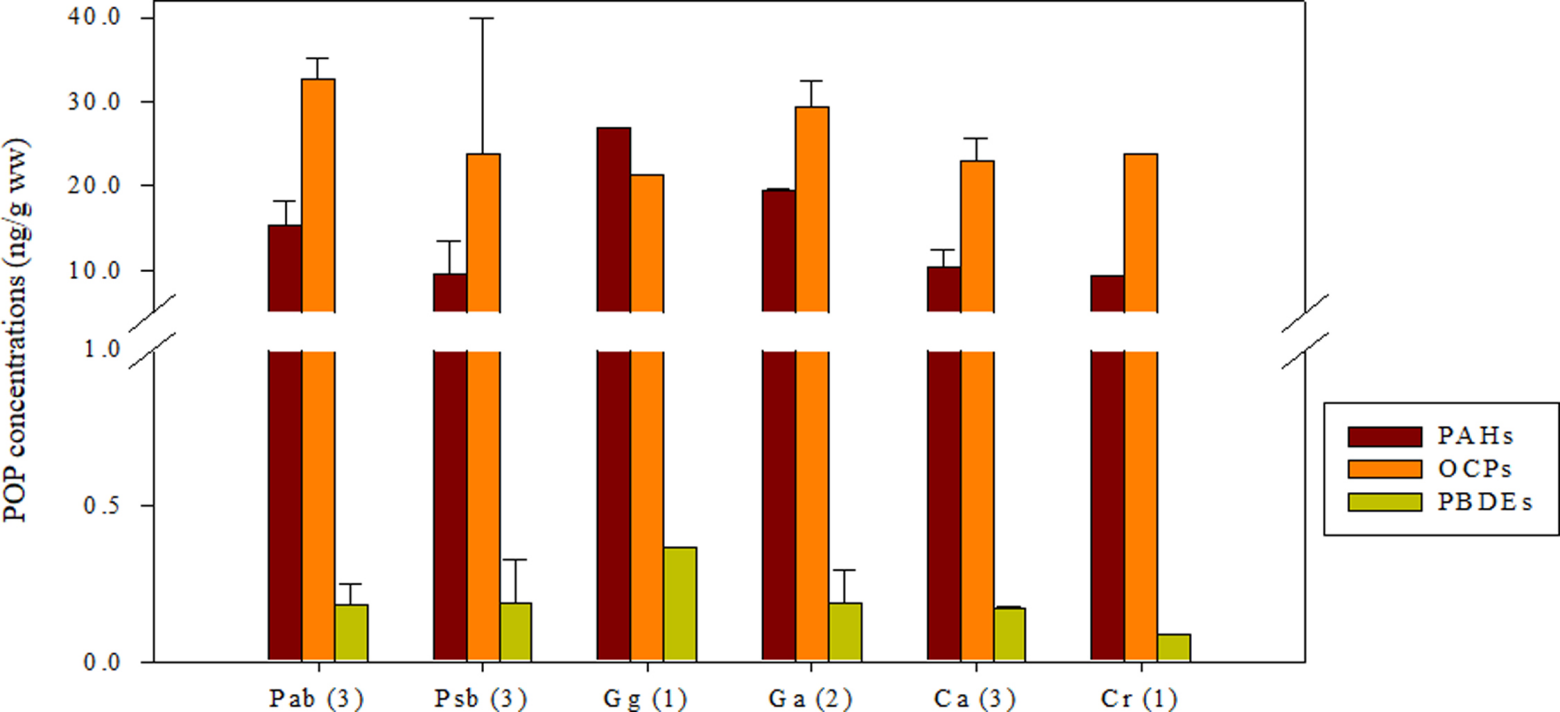
Concentration of Σ_24_PAH, Σ_11_OCP (A) and Σ_17_PBDE (B) among the six species of notothenioid fish.

All analyzed samples contained detectable levels (above the method detection limit) of the target POPs (Table 1). Fig 2 presents the fluctuation in concentration of each POP among the six species of notothenioid fish. The highest levels of total OCPs (Σ_11_OCP) was found in *Pagothenia borchgrevinki* (Pab) and the highest level of PAHs (Σ_24_PAH) and PBDEs (Σ_17_PBDE) were found in *Gobionotothen gibberifrons* (Gg). Σ_24_PAH, Σ_11_OCP, and Σ_17_PBDE in fish muscles were 6–27 ng/g ww (wet weight), 6–39 ng/g ww, and 0.1–0.4 ng/g ww, respectively. Both PAH and OCP levels in notothenioid fish were more than 20 times greater than PBDE levels. In this study, the levels of PAHs and OCPs in the analyzed samples reach those found in marine organisms from other regions of the world, while the PBDE concentrations were relatively low and comparable to those found in marine organisms from remote zones.

Since industrialization, abundant anthropogenic PAHs have been produced and distributed in the environment, and the continued transport and input of these contaminants into the Antarctic may cause increasing trends of contaminant in the polar environment. During the past few decades, excessive use of OCPs in agriculture, especially in developing countries in the Southern Hemisphere near the Antarctic, may have caused heavy bioaccumulation of OCPs in Antarctic marine organisms. Although most OCPs have been disabled or restricted, they may still be in use in some Southern Hemisphere countries, and inventory data are incomplete or not available [27]. Over the past several decades, PBDEs were widely used in the production of flame retardants [28] and added in a variety of consumer goods. Due to its hormone-disrupting effects, in particular estrogen and thyroid hormones, in both wildlife and human, PBDEs were termed as emerging pollutants, and a number of actions were executed to ban industrial production and limit the exposure and release of PBDEs in many countries, especially in the US and the European Union. Compared to the levels of PAHs and OCPs found in Antarctic notothenioid fish, the evidence of low concentrations of PBDEs (Fig 2) might be due to these restrictions, indicating the potential efficacy of the industrial production restriction and actions such as the Stockholm Convention, a treaty to control and phase out major POPs.

**Fig 3:**
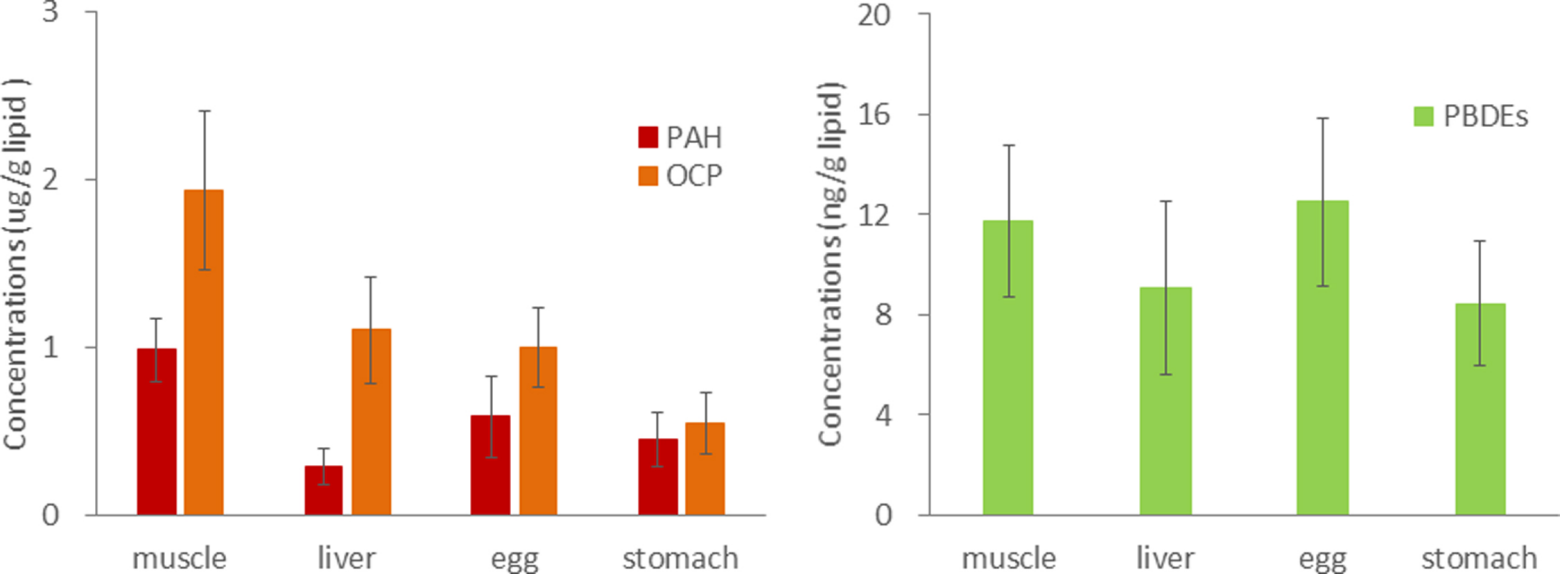
POP concentrations in muscle, liver, egg, and stomach of fish were normalized by lipid content of each tissue.

POP concentrations (based on ng/g ww) in livers and eggs of the fish samples were relatively high, probably due to the higher lipid content of these tissues. Thus, to compare the POP bioaccumulation in different tissues of the fish, the POP concentrations in muscle, liver, egg, and stomach were normalized by the lipid content of each tissue (ng/g lipid; Fig 3). Theoretically, the lipid-normalized POP concentrations are expected to be fairly constant and based on their lipophilicity. However, Fig 3 shows that POPs accumulated in muscle lipids more than in liver lipids, suggesting that POPs in the liver, especially for PAHs and OCPs, may be metabolized more readily. In comparison to fish that mainly feed on krill, higher concentrations of POPs were found in hepatic tissue than in muscle tissue in some Antarctic species of fish that feed on benthic organisms [29]. Borshesi et al (2008) [30] also found that POP levels were higher in *Trematomus bernacchii* (a benthic species) than in *Chionodraco hamatus* (inhabits the continental shelf). The bioaccumulation of various classes of POPs in the tissues of Antarctic fish may be attributable to ecological differences rather than the physiochemical properties of POPs. Additional factors, such as differences in toxicant metabolism rates and selective metabolism of various POPs, may also play an important role in defining long term chemical bioaccumulation patterns in Antarctic fish species.

The POP levels in most Antarctic invertebrates were generally greater than in fish (Table 2), suggesting that specific biogeochemical processes rather than trophic transfer, were involved in the transport, exposure, and absorption for each group of contaminants. Many studies also pointed out that certain species of aquatic invertebrates experience higher bioaccumulation of POPs than fish in the same waters [31, 32, 33]. Due to tiny body sizes, invertebrates can be modeled as fat spheres with larger surface area-to-volume ratios that can absorb non-polar compounds like POPs [34]. Some invertebrates such as amphipods are detritivores, and may accumulate higher concentrations of POPs by via contaminated detritus [35]. Additionally, invertebrate metabolic capacity may be lower than those of fish [36].

**Table 2:** Concentrations of POPs (ng/g wet weight) in Antarctic fish muscle and invertebrate samples.

Species	A*	N	Σ_24_PAH	Σ_11_OCP	Σ_17_PBDE	Σ_11_PCB
**Fishes**						
*Chaenocephalus aceratus*	Ca	3	10.4 (8.4–12.5)	23.0 (20.0–25.5)	0.2 (0.2–0.2)	23.0 (20.0–25.5)
*Chionodraco rastrospinosus*	Cr	1	9.5	23.8	0.1	23.8
*Gobionotothen gibberifrons*	Gg	1	26.8	21.3	0.4	21.3
*Gymnodraco acuticeps*	Ga	2	19.4 (19.3–19.6)	29.3 (27.1–31.6)	0.2 (0.1–0.3)	29.3 (27.1–31.6)
*Pagothenia borchgrevinki*	Pb	3	15.4 (12.8–18.4)	32.7 (30.5–35.2)	0.2 (0.1–0.3)	32.7 (30.5–35.2)
*Pseudotrematomus bernacchii*	Psb	3	9.5 (6.3–14.0)	23.8 (6.6–38.8)	0.2 (0.1–0.3)	23.8 (6.6–38.8)
**Invertebrates**						
Gammaridea gn. sp.	Gam	2	50.4 (46.3–54.5)	38.9 (34.3–43.5)	0.4 (0.3–0.4)	38.9 (34.3–43.5)
*Euphausia superba*	Es	2	67.4 (62.3–72.4)	4.4 (3.5–5.3)	1.3 (0.8–2.2)	4.4 (3.5–5.3)
*Nacella concinna*	Nc	1	21.5	15.1	0.3	15.1
*Amauropsis* sp.	As	1	752.8	123	124.9	123
*Yoldia sp*.	Ys	2	35.85 (28.4–43.3)	22.15 (20.4–23.9)	1.3 (0.8–1.8)	22.15 (20.4–23.9)
*Ophionotus victoriae*	Ov	2	7.4 (5.4–9.4)	13.6 (10.7–16.6)	0.1 (0.1–0.2)	13.6 (10.7–16.6)
*Sterechinus neumayeri*	Sn	2	49.2 (46.1–52.4)	101.4 (92.9–110.0)	1.1 (1.0–1.1)	101.4 (92.9–110.0)
*Sterechinus* sp.	Ss	2	20.1 (14.6–25.7)	29.9 (19.3–40.5)	0.4 (0.3–0.4)	29.9 (19.3–40.5)
Salpidae gn. sp.	Sal	1	84.2	6.5	1.3	6.5

*Abbreviation

**Fig 4:**
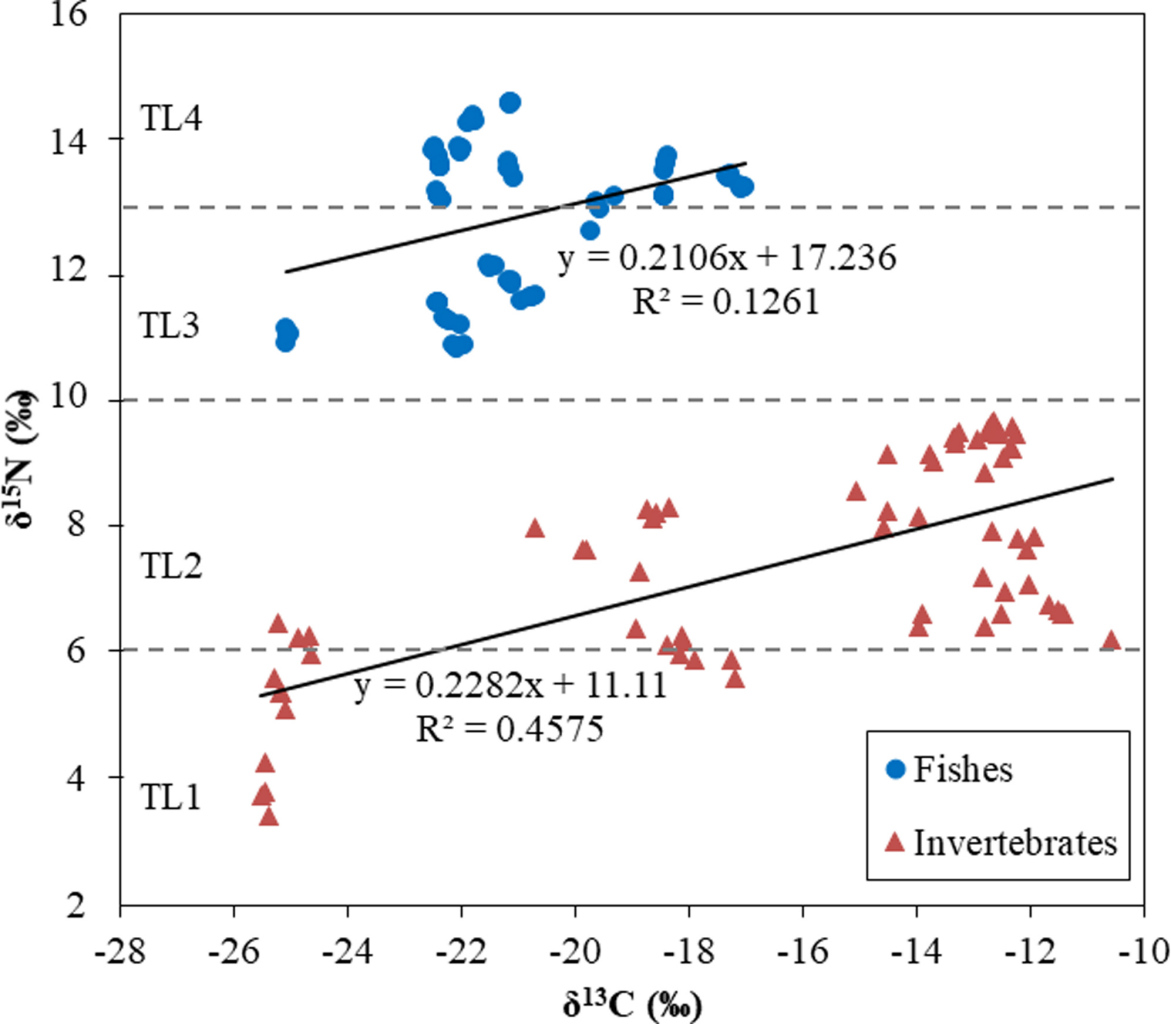
Range of stable isotope ratios of carbon (δ^13^C) and nitrogen (δ^15^N) in the Antarctic notothenioid fish and invertebrates.

Stable isotope ratios of carbon (δ^13^C) and nitrogen (δ^15^N) are showed in Fig 4. Carbon stable isotope ratios (δ^13^C) in invertebrates (-26‰ to -12‰) showed a greater range than in fish tissue (-23‰ to -17‰), due to differences in physiology and biochemistry. A large range of δ^15^N (4‰ to14‰) in fishes and invertebrates was found and seemed to be related to nitrogen uptake from different animal-derived sources leading to the classification of four trophic levels (from TL1 to TL4), which indicates that fish is ranked in the higher TL than invertebrate (Fig 4).

**Fig 5:**
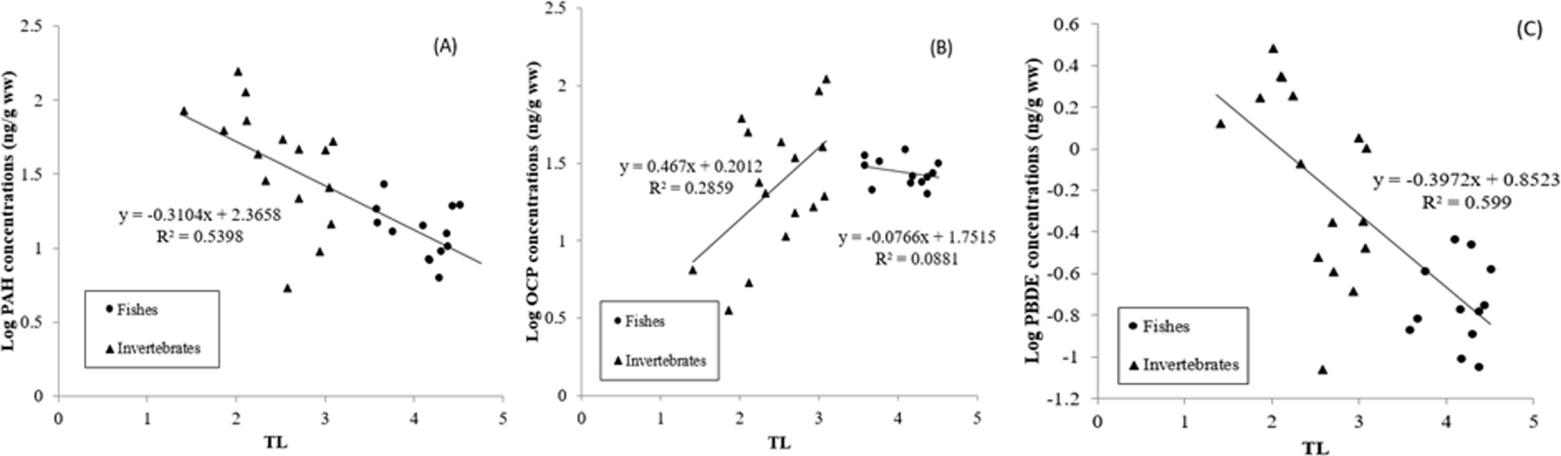
The interrelation of trophic level (TL) v.s. concentrations of log PAH (A), concentrations of log OCP (B), and concentrations of log PBDE (C).

Based on the relationship between the δ^15^N values (trophic levels) and the POP concentrations in fish and invertebrates in this study (Fig 5), there is not a clear role for biomagnification of POPs through the food chain in this research zone of the Antarctic. The negative correlation between TL and most POP concentrations in organisms (except OCPs in invertebrates) indicates that specific biogeochemical processes, rather than biomagnification, were involved in the source, transport, exposure, and absorption of POP contaminants. POP availability in the Antarctic environment and specific ecological features such as short food chain may play an important role in POP bioaccumulation.

## Conclusion

Banned pesticides such as HCB and *p*,*p*′-DDE, and other legacy and ongoing pollutants such as PBDEs and PAHs, were measured in fish and invertebrate samples collected at Chun-Shun research station adjacent waters in eastern Antarctic and found to reach levels comparable to those from other areas of the world. The POPs accumulated more in fish muscle than in the liver, suggesting that liver metabolism may reduce the bioaccumulation of POPs. POP concentrations in Antarctica fish and invertebrates negatively correlated with trophic level, suggesting that specific biogeochemical processes may be involved in the sources, transport, exposure, and absorption for each POP contaminant. Biological variation among species, habitat interaction, diet, metabolism, and growth dilution may also affect the particularly unusual patterns of POP bioaccumulation in notothenioid fish in the Antarctic.

## Supporting information

S1 Table Analyzed species of biota samples. Identification information of the analyzed biota samples (fishes and invertebrates).
(PDF)
