# Supplementary material for: Persistent organic pollutants in Antarctic notothenioid fish and invertebrates associated with trophic levels
Source: PLoS One. 2018 Apr 11;13(4):e0194147. doi: 10.1371/journal.pone.0194147 (PMC5894976; doi:10.1371/journal.pone.0194147)
Supplement: S1 Table — Identification information of the analyzed biota samples (fishes and invertebrates). (PDF) [file pone.0194147.s001.pdf]

Table S1. Identification information of the analyzed biota samples (fishes and invertebrates).

| | |
|---|---|
| Fishes | *Chaenocephalus aceratus* (Lönnberg, 1906)<br>*Chionodraco rastrospinosus* DeWitt & Hureau, 1979 |
| | *Gobionotothen gibberifrons* (Lönnberg, 1905) |
| | *Gymnodraco acuticeps* Boulenger, 1902 |
| | *Pagothenia borchgrevinki* (Boulenger, 1902) |
| | *Pseudotrematomus bernacchii* (Boulenger, 1902) |
| Invertebrates | Gammaridae gn. sp. |
| | *Euphausia superba* Dana, 1850 |
| | *Nacella concinna* (Strebel, 1908) |
| | *Amauropsis* sp. |
| | *Yoldia* sp. |
| | *Ophionotus victoriae* Bell, 1902 |
| | *Sterechinus neumayeri* (Meissner, 1900) |
| | *Sterechinus* sp. |
| | Salpidae gn. sp. |
